# Cultural adaptation of the mental health first aid guidelines for assisting a person at risk of suicide for Sri Lanka: a Delphi expert consensus study

**DOI:** 10.1186/s12888-021-03486-7

**Published:** 2021-09-24

**Authors:** Amila Chandrasiri, Madhawee Fernando, Madhubhashinee Dayabandara, Nicola J. Reavley

**Affiliations:** 1 Centre for Mental Health, Melbourne School of Population and Global Health, University of Melbourne, Parkville, Victoria 3010 Australia; 2 Ministry of Health, Colombo, Sri Lanka; 3 Department of Psychiatry, Faculty of Medicine, University of Colombo, Colombo, Sri Lanka

**Keywords:** Suicide, Mental health first aid (MHFA), Cultural adaptation, Delphi study, Sri Lanka

## Abstract

**Background:**

Approximately 3000 people die by suicide each year in Sri Lanka. As family and friends may play a role in supporting a person at risk of suicide to get appropriate help, there is a need for evidence-based resources to assist with this. The aim of this study was to culturally adapt the existing English-language mental health first aid guidelines for helping a person at risk of suicide to the Sri Lankan context.

**Methods:**

A Delphi expert consensus study was conducted, involving mental health professionals and consumers (people with lived experience) and caregivers, who were identified by purposive and snowball sampling methods. Participants were recruited from a wide variety of professional roles and districts of Sri Lanka in order to maximize diversity of opinion. The original questionnaire was translated into Sinhala and participants were requested to rate each item according to the importance of inclusion in the guidelines.

**Results:**

Data were collected over two survey rounds. Altogether, 148 people participated in the study (130 health professionals and 18 consumers). A total of 165 items were included in the final guidelines, with 153 adopted from the guidelines for English-speaking countries and 12 generated from the comments of panellists.

**Conclusions:**

The adapted guidelines were similar to the English-language guidelines. However, new items relating to the involvement of family members were included and some items were omitted because they were not considered appropriate to the Sri Lankan context (particularly those relating to explicit mention of suicide). Further research is warranted to explore the use of these guidelines by the Sri Lankan public, including how they may be incorporated in Mental Health First Aid training.

**Supplementary Information:**

The online version contains supplementary material available at 10.1186/s12888-021-03486-7.

## Background

Suicide is an important public health issue worldwide, with nearly 800,000 people dying by suicide every year [1]. In 2016, suicide accounted for 1.4% of all deaths worldwide and was the 16th leading cause of death [2]. Though accurate figures are not available, it is estimated that for each adult who dies by suicide, twenty more may have made an attempt [3]. In 1995, Sri Lanka had the highest female suicide rate in the world (at 47 per 100,000 people) [4]. Recommendations suggested in the 1997 report of the Presidential committee on the prevention of suicides were implemented and have contributed to a decline in suicide mortality from 60 per 100,000 people in 1995 to 18.5 per 100,000 in 2011 [5]. Regulatory controls on the importation and sales of highly toxic pesticides were the most important contributing factor to this reduction [6]. Despite this decline, marked fluctuations in suicide rates in Sri Lanka have been seen and suicide remains a significant problem, with 4523 deaths by suicide in 2017 and a mortality rate of 19.8 per 100,000 people [7]. Recent data show that lower socioeconomic position (including having fewer assets, insecure/low-income jobs and lower levels of education) is associated with an increased risk of suicide [8] Other community risk factors include community ‘problem’ alcohol use and living in households with alcohol misuse (for women), while living in multigenerational households appeared to be protective [9].

In Sri Lanka, a range of health professionals provide services to people at risk of suicide, including both the preventive and curative sectors [10]. The curative sector covers hospitals, which provide both in-patient and out-patient services while the preventive sector covers prevention and promotion services. In the mental health service model operating in Sri Lanka a wide range of professionals provide services in the curative sector. These include specialist psychiatrists, psychiatric medical officers (doctors), psychiatric nurses, psychologists and social workers. In the public health sector health workers delivering mental health services include specialist community physicians, medical officers (public health) and midwives.

However, the majority of resources are allocated to the curative sector [11] and the prevention and promotion components of the mental health service system are less well-developed, with numbers of trained mental health professionals remaining inadequate to meet the mental healthcare needs of the Sri Lankan population [12]. Sri Lanka’s mental health policy (2005–2015) stressed the need to implement a comprehensive community-based, service structure and also emphasized the importance of active involvement of communities and families, both in preventing mental health problems and ensuring better access to mental health services [11]. Given the evidence that family members, friends and others in a person’s social network can facilitate help-seeking, empowering the community with knowledge and skills to help detect problems early and encourage people at risk to seek help, may be helpful in suicide prevention [13, 14].

The Mental Health First Aid (MHFA) training program was developed in response to the demand for interventions to meet this need. It aims to train members of the public in how to assist someone who is developing a mental illness or in a mental health crisis situation (e.g. suicide). MHFA was originally developed in Australia in 2000 [15] and has now spread to more than 25 other (mostly high-income, English-speaking) countries. Over 4 million people have been trained globally [16]. Evidence suggests that MHFA training improves mental health knowledge, reduces stigma and leads to the provision of appropriate support for people with mental health problems [17, 18]. However, most of the studies of MHFA have been conducted in high-income countries and its suitability for use in low and middle-income countries (LMICs) is unclear [15].

MHFA training is informed by a series of Delphi expert consensus studies involving health professionals and people with lived experience [19]. However, most such studies have involved participants in high-income countries, although guidelines for India and the Philippines have been developed [20, 21]. In 2016 a Delphi study was conducted to develop suicide first-aid guidelines for Sri Lanka [22]. However, the questionnaire was only administered in English to health professionals and the views of people with lived experience or lower levels of education or were not captured. Therefore, the aim of the study was to use the Delphi expert consensus method with English and Sinhala-speaking Sri Lankan health professionals and people with lived experience to culturally adapt the mental health first aid guidelines for Sri Lanka.

## Methods

### The Delphi method

The Delphi method is an approach to transforming the opinions of individual experts into group consensus. It has been used in multiple fields including mental health research [20, 23–25]. In this study, the Delphi method was used to obtain consensus between mental health professionals and people with lived experience on appropriateness of statements to be included in the guidelines on helping a person at risk of suicide in Sri Lanka.

Study procedures included the following stages; (1) translation of English language questionnaire to Sinhala, the national language of Sri Lanka, (2) panel member identification and recruitment; (3) data collection over 2 rounds of survey; (4) data analysis and (5) guidelines development.

### Translation of the English language questionnaire to Sinhala

The English language questionnaire (consisting of the items that were included in the final guidelines) was translated into Sinhala by a health professional. During this process, minor changes were made to some statements to make them more appropriate to the Sri Lankan health system and cultural context, e.g., emergency ambulance services which can be summoned by public are not available everywhere in Sri Lanka so this item was replaced with ‘calling Suwasariya (1990) ambulance service or organizing an alternative means to take the person to hospital’. Similarly ‘to see a GP’ was replaced with ‘to see the family doctor’, as this term is more common in Sri Lanka [26]. Because of the assumption that all the health workers can read and understand English and most of the consumers within the study areas can comprehend either Sinhala or English, Tamil translation was not done although that is the second most commonly used language in Sri Lanka.

The round 1 questionnaire comprised 168 statements categorized under 8 sections. Participants were asked to rate each item according to its importance for inclusion in the Sri Lankan guidelines on a five-point Likert scale with response options of, essential, important, depends/don’t know, unimportant and should not be included. The questionnaire also contained questions about socio-demographic characteristics, professional status and experience in mental health service provision (for health professionals).

### Identification and recruitment of participants

Two groups of participants were recruited into the study: (1) Mental health professionals, (2) People with lived experience and caregivers (also referred to as consumers). Mental health professionals met eligibility criteria if they had been involved in providing mental health services for at least 2 years’ in either the state or private curative and preventive sectors. Inclusion criteria for consumers were as follows: (1) They had at least 1 year’s lived experience after an attempt of suicide; or (2) Or 1 years’ experience in caring for a person at risk of suicide.

Purposive and snowball sampling approaches were used to recruit professionals and people with lived experience. In order to maximize diversity of opinion (which is important for Delphi expert consensus studies) [27], we aimed to recruit participants from a wide variety of professional roles and from five different administrative districts across four provinces. This included tertiary and secondary level specialized Mental Health Units with in-patient care, primary level Mental Health Units providing out-patient and follow up care and public health institutes providing community- level care.

After identification of settings for recruitment, approval was obtained from relevant administrative authorities. For specialized mental health units (secondary and tertiary care), approval was obtained from the Director of the institute. One of the authors (AC) visited the units and directly approached participants, explained the purpose of the study and offered paper questionnaires. Participants were then free to decide whether or not to complete these. For primary level mental health units and public health institutes, the respective Regional Directors were approached for permission. AC visited these facilities during their monthly review meetings and directly approached participants.

In order to recruit people with lived experience, for each setting, a coordinator (typically a clinic nurse) was identified. This coordinator explained the purpose of the study and invited those eligible and interested in participating to attend a session in which the questionnaires were distributed and administered.

### Data collection

In each setting, a short introduction was given to all participants, in which they were instructed to rate how important the statements were to be included in the guidelines. Health professionals were given the choice of whether to complete the survey in Sinhala or English, while consumers were given Sinhala questionnaires. Participants were also requested to add comments modifying existing statements or to suggest new items to be included. In recompense for their time, participants were given a gift voucher valued at Sri Lankan Rs 1500 for completing at least the Round 1 survey.

### Data analysis

Statements were immediately included in the guidelines if they were endorsed by ≥80% of members in both panels as either essential or important. Statements were re-rated in the following round if they were rated as essential or important by 70–79% of either panels but were excluded if they were rated as essential or important by less than 70% of one panel.

Comments and suggestions from participants were refined, sorted and translated into English by one of the authors (AC) and then reviewed by authors, NR, AC and MF. New ideas were written into statements and included in the Round 2 questionnaire. The Round 2 questionnaire comprised 17 Items selected for re-rating based on the above-mentioned criteria and 14 newly generated items (a total of 31 items).

Participant socio-demographic characteristics, professional status and experience in mental health service provision were analyzed using descriptive statistics. Endorsement levels for each item were also calculated. The correlation between the endorsement rates of two panels of professionals and consumers was assessed using Spearman’s correlation coefficient. Analysis was done by SPSS Version 16.0 statistical software.

### Guidelines development

Endorsed statements (i.e. those being rated as either essential or important by ≥80% of both panels) from both rounds were compiled. The Sinhala guidelines were developed by writing the list of endorsed statements into sections of connected text. Statements were amalgamated when possible. The language was changed in certain items to clarify meaning. The draft was then circulated to a panel of Sinhala speaking experts and non-health professionals for final review. Their inputs were included, and final guidelines were drafted.

## Results

### Expert panel formation

The total number of participants in the study was 148 (130 mental health professionals and 18 consumers). The socio-demographic characteristics of all participants are shown by panel in Table 1.

**Table 1:** The socio-demographic characteristics of all participants

Variable	Mental health professionals		Consumers	
	Frequency (***n*** = 130)	Percentage (%)	Frequency (***n =*** 18)	Percentage (%)
**Gender**				
Male	30	23.1	6	33.3
Female	100	76.9	12	66.7
**Age category**				
18–34	4	3.1	2	11.1
35–44	25	19.2	10	55.6
45–54	49	37.7	3	16.7
55–64	42	32.3	3	16.7
65 and above	10	7.7	0	0.0
**Highest educational qualification**				
Primary school	0	0.0	2	11.1
Secondary school / high school	9	6.9	12	66.7
Technical diploma	56	43.1	4	22.2
Bachelor’s degree	27	20.8	0	0.0
Master’s degree	6	4.6	0	0.0
Doctorate (Higher degree by research) or PhD	8	6.2	0	0.0
Other	18	13.8	0	0.0
Missing	6	4.6	0	0.0
**Principal area of practice**				
Mental health clinician- specialist	9	6.9	NR	NR
Mental health clinician	24	18.5	NR	NR
Psychiatric social worker	8	6.2	NR	NR
Psychologist	3	2.3	NR	NR
Midwife	22	16.9	NR	NR
Nursing Officers (Mental Health)	56	43.1	NR	NR
Missing	8	6.2	NR	NR
**Principal setting of practice / affiliation**				
Government hospital	73	56.2	NR	NR
Community Mental Health Service	25	19.2	NR	NR
Educational facility	3	2.3	NR	NR
Other	10	7.7	NR	NR
Missing	19	14.6	NR	NR
**Years worked in the principal area of practice/ as consumer**				
2-4	41	31.5	10	55.6
more than 4	74	56.9	2	11.1
Missing	15	11.5	0	0.0
**Lived experience Vs Caregiver status among consumers**				
Consumers with lived experience	NR	NR	9	50.0
Consumers who were care givers	NR	NR	9	50.0

Higher female representation was noted in both groups as 100 (76.9%) professionals and 12 (66.7%) consumers were females. Among the professionals, most were aged between 45 and 54 years (37.6%), while in the consumer group, most were aged between 35 and 44 years (55.6%). Among professionals, the single largest category was that of nurses from mental health units (*n* = 56, 43.1%). There were 33 doctors including specialist psychiatrists, postgraduate qualified mental health doctors, diploma qualified mental health doctors and doctors who deliver community mental health services. Among professionals, most (56.2%, *n* = 73) were employed in government hospitals. Among consumers, 50% of people had their own lived experiences while the remaining 50% were caregivers. Among the mental health professionals who took part in the first round, 98 (66.2%) were retained in round 2, while the retention rate among consumers was 38.8% (*n =* 7).

### Ratings of statements

**Fig. 1:**
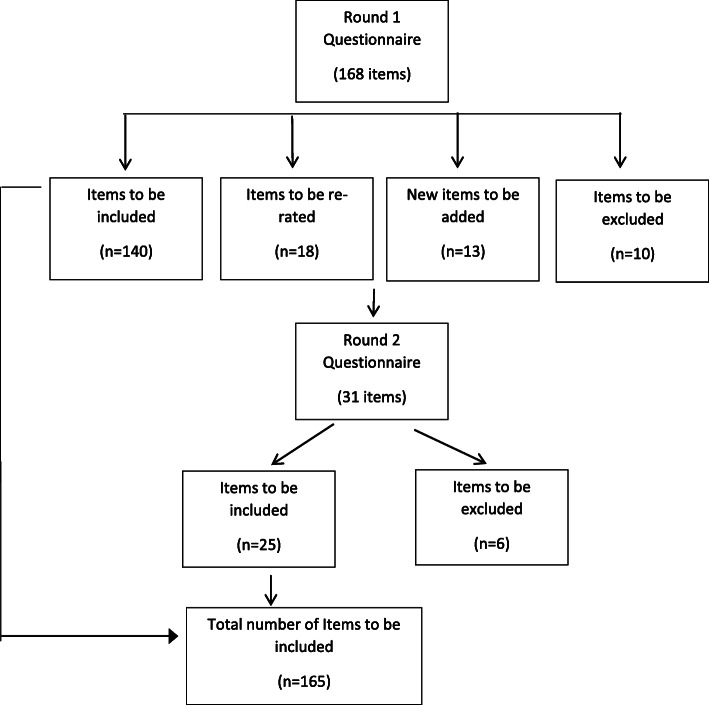
Overview of the study rounds

All the items were categorized into 3 groups based on the responses given by participants. Responses marked as either essential or important were considered as endorsed. Items which had more than 80% of endorsements in both panels were immediately included in the final guidelines (*n* = 140). Items which had endorsement rates between 70 - 79% in either of the panels (*n* = 18) were re-rated in Round 2, while items which had an endorsement rate of less than 70% from either of the panels (*n* = 10) were omitted. Based on the comments made by participants in the 1st round, a new set of items were prepared and included in Round 2 (*n* = 13). Thus, a total of 30 items were included for the 2nd round. After Round 2, items with an endorsement rate of less than 70% in either of the panels (*n* = 6) were omitted and the rest of the items were included (*n* = 25). After this, 165 items were included in the final version of the guidelines (See Fig. 1). A complete list of items and ratings is provided in Additional file 1.

A significant correlation was noted between the endorsements of the two panels in Round 1 (Spearman’s correlation coefficient *r =* 0.50 (*p* < 0.001). The correlation coefficient for Round 2 was not calculated due to the unequal drop-out of the two panels.

### Differences between professional and lived experience panels

Compared to professionals, consumers gave lower ratings of endorsement than health professionals for items focused on exploring the suicidal plan of the person by first aider; “*The first aider should ask the suicidal person if they have a plan for suicide”* (77.8% Vs 88.5%)*,* “*The first aider should ask the suicidal person how they intend to suicide i.e. ask them direct questions about how, when and where they intend to suicide”* (77.8% Vs 85.4%) and *“The first aider should ask the suicidal person if they have decided when they will carry out their plan”* (77.8% Vs 84.5%) ‘*The first aider should ask the suicidal person how they are feeling right now’*). Professionals endorsed this (96.9%) while consumers gave lower ratings (77.8%). A similar contradiction was noted for the statement was noted for the statement ‘*Discuss with the suicidal person what actions they should take to get help’* (with endorsement rates of 66.7% vs 87.5%).

### Differences between the English-language and Sri Lankan guidelines

Compared to the guidelines for English-speaking countries, 14 items were excluded, and 12 new ones were added in the adapted guidelines. Significant omissions were the items which referred to the explicit use of the term ‘suicide’ and those related to discussing option for help seeking with the person. Significant additions were the items related to involving family.

## Discussion

The aim of this study was to culturally adapt the MHFA guidelines for helping a person at risk of suicide used in English-speaking countries to Sri Lanka. A two-round Delphi expert consensus study was conducted, involving both mental health professionals and people with lived experience. Participants were recruited from a wide variety of professional roles and several districts to ensure diversity of opinion and were asked to rate the importance of including actions from the English-language guidelines and were also asked to suggest new items. While the similarity between the English-language and Sinhala guidelines was considerable, some important differences were found, pointing to important issues for future use of the adapted guidelines.

### Similarities and differences between the English-language and Sri Lankan guidelines

The high endorsement rate of items in the round 1 (83.3%, 140 out of 168) indicated a wide agreement on providing mental health first aid to people at risk of suicide between English and Sinhala versions. A similarly high level of agreement (92%) was seen in a recent study to re-develop the suicide first aid guidelines for China [28].

New items included those relating to the importance of the influence of family, both in terms of risk and protective factors for suicide. Items, *“If the person has a plan for suicide, the first aider should tell the person’s close family members about this”* and “*If the first aider is not a family member, they should ask family members of the person about their symptoms”* were endorsed. This is likely to reflect the fact that many people in Sri Lanka live in extended families with higher levels of social connectedness than is often seen in Western countries [29]. Thus, the practical and emotional support provided by family members and relatives is likely to have a positive effect on mental health and be protective against suicide [30] and family members may play a greater role in assisting a person to cope with daily stressors [31]. However intolerance of interpersonal distress and communication difficulties with family members have been identified as major risk factors for suicide and suicide attempts in Sri Lanka, particularly in women [32, 33]. It is also acknowledged that interpersonal conflicts and domestic violence also increase the risk of suicide and involving family may help to address these risks [34].

Using terms like ‘suicide’ and ‘died by suicide’ was rejected by both panels possibly due to stigmatizing attitudes towards suicide and a reluctance to address the topic directly [35]. Very low rates of agreement were noted for the item *“The first-aider should demonstrate appropriate language when referring to suicide by using the terms ‘suicide’ or ‘died by suicide”.*

The item in the English guidelines: “*The first aider should not try to take on the suicidal person’s responsibilities*” was rejected by both groups of participants. This may be due to the greater level of involvement of family in the care of people with mental health conditions, whereas in high-income countries, there is greater emphasis on the recovery approach, in which more responsibility is vested in the person [36]. It is possible that this also underlies the differences in ratings of the items ‘*Discuss with the suicidal person what actions they should take to get help’* (with endorsement rates of 64.7% (consumers and carers vs 94.4% (professionals)), with people with lived experience preferring to take a greater level of control rather than discussing this with the person.

### Differences between professional and lived experience panels

It is likely that the round 1 differences between the professional and lived experience panels, which mostly focused on items relating to asking about suicide, reflect low levels of mental health literacy and a reluctance to ask directly about suicide due to a commonly held perception that enquiring about suicidality can increase the risk of suicide despite evidence that it is beneficial [37] and may lead to improvements in mental health in treatment-seeking populations [38]. It is only relatively recently that beliefs about the risks of asking directly about suicide have begun to shift as a result of suicide prevention campaigns and other interventions [38]. It is likely that health professionals are more aware of this evidence and therefore are more likely to endorse such statements. This is also reflected in the relatively lower levels of correlation between the health professional and lived experience panels in Sri Lanka compared to the English language guidelines, which are also similar to the study done in China [28]. The results of this study point to the need to for similar efforts to explore and address attitudes in the general population in Sri Lanka.

In relation to the item about involving police in situations in which the person is carrying a weapon, professionals showed a lower level of agreement than consumers (with 70.2% vs 93.8% of endorsement). Police involvement in incidents involving mentally ill patients has increased with the growth in community psychiatry [39], and it is possible that the differences in ratings are due to a perception among the general public, that people with mental health problems are more likely to be violent [40].

### Considerations for future use of the adapted guidelines

This study involved obtaining the views and opinions of mental health professionals and people with lived experience in Sri Lanka to modify the guidelines for assisting a person at risk of suicide. Findings of the study had revealed a high degree of endorsement, confirming the overall relevance of the guidelines. The guidelines can be used as a standalone product and may also be used to inform a culturally adapted MHFA training program for Sri Lanka. Such a program may also help to improve mental health literacy and reduce stigma in the general population. However, trialing and adaptation of the program to be suitable for implementation in the Sri Lankan health system are necessary for any potential gains to be achieved.

### Strengths and limitations

Strengths of the study involved the wide range of health professionals, enabling diverse views to be obtained. A further strength was the inclusion of people with lived experience, although the relatively small number means that one person’s rating may have had a relatively large impact on the results. Moreover, due to the recruitment strategy, these were only people who had been in contact with health services and the views of people who had not been in contact with services were not captured. While common in many high-income countries, inclusion of people with lived experience remains relatively rare in contexts such as Sri Lanka. Conducting the study in Sinhala was also a strength as it allowed for the inclusion of people with lower levels of education who may not have been able to complete the study in English. This was reinforced by the fact that most of the health professional participants preferred Sinhala as the language of choice. Comparisons with the English-language guidelines are limited by potential differences in understanding terminology in English and Sinhala. However, as the guidelines do not use highly technical language, we don’t believe this to be a significant limitation.

A further limitation is that the study was not conducted among Tamil-speaking participants, largely for logistical reasons. In an effort to overcome this weakness, the final guidelines will be translated into Tamil. Finally, the attrition rate between rounds 1 and 2 is a limitation, particularly in the case of the lived experience panel, who were more difficult to contact in the 2nd round.

## Conclusions

A Delphi expert consensus study involving local mental health professionals and people with lived experience was used to culturally adapt the Mental Health First Aid guidelines to assist a person at risk of suicide for Sri Lanka. While there were many similarities to the guidelines for English-speaking countries, several items were omitted, particularly those relating to asking about suicide, exploring the suicidal plan and involving police in situations where the person had a weapon. The adapted guidelines included several new items, particularly those relating to involving family members. Further research on the dissemination and uptake of the guidelines as well as their use in informing MHFA training is needed.

## Supplementary Information

**Additional file 1.** Supplementary information accompanies this paper: 1. Statements that were presented to the panels and their ratings across 2 rounds of the survey.

## Data Availability

The data supporting our findings is attached as the Additional file, which contains all the statements that were presented to the panels and their endorsement rates.

## Abbreviations

MHFA: Mental Health First Aid;
LMICs: Low- and middle-income countries

## References

[1] World Health Organization. Suicide Data 2020 [cited 2020 09th March 2020]. Available from: https://www.who.int/mental_health/prevention/suicide/suicideprevent/en/.

[2] World Health Organization (2019). World Health Statistics Overview.

[3] Lonnquist JK (2009). Epidemiology and causes of suicide.

[4] Sri Lanka Sumithrayo (2010). Statistics and Data of Suicides: Sri Lanka Sumithrayo.

[5] Knipe DW, Chang S-S, Dawson A, Eddleston M, Konradsen F, Metcalfe C, Gunnell D (2017). Suicide prevention through means restriction: impact of the 2008-2011 pesticide restrictions on suicide in Sri Lanka. PLoS One.

[6] Rice SM, Goodall J, Hetrick SE, Parker AG, Gilbertson T, Amminger GP, Davey CG, McGorry PD, Gleeson J, Alvarez-Jimenez M (2014). Online and social networking interventions for the treatment of depression in young people: a systematic review. J Med Internet Res.

[7] Institute for Health Metrics and Evaluation. Global Burden of Disease Collaborative Network. In: Global Burden of Disease Study 2016 (GBD 2016) Results, vol. 2017: Institute for Health Metrics and Evaluation Seattle.

[8] Knipe DW, Gunnell D, Pieris R, Priyadarshana C, Weerasinghe M, Pearson M, Jayamanne S, Dawson AH, Mohamed F, Gawarammana I, Hawton K, Konradsen F, Eddleston M, Metcalfe C (2017). Is socioeconomic position associated with risk of attempted suicide in rural Sri Lanka? A cross-sectional study of 165 000 individuals. BMJ Open.

[9] Knipe DW, Gunnell D, Pearson M, Jayamanne S, Pieris R, Priyadarshana C, Weerasinghe M, Hawton K, Konradsen F, Eddleston M, Metcalfe C (2018). Attempted suicide in Sri Lanka - an epidemiological study of household and community factors. J Affect Disord.

[10] Perera S, Nieveras O, de Silva P, Wijesundara C, Pendse R (2019). Accelerating reforms of primary health care towards universal health coverage in Sri Lanka. WHO South East Asia J Public Health.

[11] Fernando N, Suveendran T, de Silva C (2017). Decentralizing provision of mental health care in Sri Lanka. WHO South East Asia J Public Health.

[12] Weerasundera R (2010). Community Psychiatry in a Sri Lankan setting: Should we rush to push the boundaries?. Sri Lanka J Psychiatry..

[13] Vogel DL, Wade NG, Wester SR, Larson L, Hackler AH (2007). Seeking help from a mental health professional: the influence of one's social network. J Clin Psychol.

[14] Seneviratne HSD, Sanjeewani RMSS (2019). Demographic characteristics of suicides in Sri Lanka from 2006 to 2018.

[15] Jorm AF, Kitchener BA. Noting a landmark achievement: mental health first aid training reaches 1% of Australian adults: Taylor & Francis; 2011. 10.3109/00048674.2011.594785 21827342

[16] Jorm AF, Kitchener BA, Reavley NJ (2019). Mental health first aid training: lessons learned from the global spread of a community education program. World Psychiatry.

[17] Morgan AJ, Ross A, Reavley NJ. Systematic review and meta-analysis of Mental Health First Aid training: Effects on knowledge, stigma, and helping behaviour. PLoS One. 2018;13(5). 10.1371/journal.pone.0197102 PMC5979014 29851974

[18] Hanisch SE, Twomey CD, Szeto AC (2016). The effectiveness of interventions targeting the stigma of mental illness at the workplace: a systematic review. BMC Psychiatry.

[19] Jorm AF (2015). Using the Delphi expert consensus method in mental health research. Aust N Z J Psychiatry.

[20] Colucci E, Kelly CM, Minas H, Jorm AF, Chatterjee S (2010). Mental health first aid guidelines for helping a suicidal person: a Delphi consensus study in India. Int J Ment Heal Syst.

[21] Colucci E, Kelly CM, Minas H, Jorm AF, Nadera D (2010). Mental health first aid guidelines for helping a suicidal person: a Delphi consensus study in the Philippines. Int J Ment Heal Syst.

[22] De Silva SA, Colucci E, Mendis J (2016). Suicide first aid guidelines for Sri Lanka: a Delphi consensus study. Int J Ment Heal Syst.

[23] Armstrong G, Ironfield N, Kelly CM, Dart K, Arabena K, Bond K, Jorm AF (2017). Re-development of mental health first aid guidelines for supporting Aboriginal and Torres Strait islanders who are engaging in non-suicidal self-injury. BMC Psychiatry.

[24] Bond KS, Cottrill FA, Blee FL, Kelly CM, Kitchener BA, Jorm AF (2019). Offering mental health first aid to a person with depression: a Delphi study to re-develop the guidelines published in 2008. BMC Psychol.

[25] Ross AM, Kelly CM, Jorm AF (2014). Re-development of mental health first aid guidelines for suicidal ideation and behaviour: a Delphi study. BMC Psychiatry.

[26] Ramanayake R (2013). Historical evolution and present status of family medicine in Sri Lanka. J Fam Med Prim Care.

[27] Luo L, Wildemuth BM (2009). Delphi studies. Applications of social research methods to questions in information and library science.

[28] Lu S, Li W, Oldenburg B, Wang Y, Jorm AF, He Y, Reavley NJ (2020). Cultural adaptation of the mental health first aid guidelines for depression used in English-speaking countries for China: a Delphi expert consensus study. BMC Psychiatry.

[29] Caldwell B (1996). The family and demographic change in Sri Lanka. Health Transit Rev.

[30] Sharaf AY, Thompson EA, Walsh E (2009). Protective effects of self-esteem and family support on suicide risk behaviors among at-risk adolescents. J Child Adolesc Psychiatr Nurs.

[31] Andriessen K, Krysinska K. A psycho-educational perspective on family involvement in suicide prevention and postvention. Suicide. 2016:333–44. 10.1093/med/9780198717393.003.0032.

[32] Rajapakse T, Griffiths KM, Christensen H, Cotton S (2015). Non-fatal self-poisoning in Sri Lanka: associated triggers and motivations. BMC Public Health.

[33] Marecek J (2006). Young women’s suicide in Sri Lanka: cultural, ecological, and psychological factors. Asian J Couns.

[34] Chandrasiri P, Lokubalasuriya A, Hettiarachchi R (2018). Epidemiology of attempted suicides in Southern Sri Lanka. Galle Med J..

[35] Oexle N, Herrmann K, Staiger T, Sheehan L, Rüsch N, Krumm S (2018). Stigma and suicidality among suicide attempt survivors: a qualitative study. Death Stud.

[36] Stratford A, Brophy L, Beaton T, Castle D (2013). Recovery, medication and shared responsibility in mental health care. Australas Psychiatry.

[37] Mathias CW, Michael Furr R, Sheftall AH, Hill-Kapturczak N, Crum P, Dougherty DM (2012). What’s the harm in asking about suicidal ideation?. Suicide Life Threat Behav.

[38] Dazzi T, Gribble R, Wessely S, Fear NT (2014). Does asking about suicide and related behaviours induce suicidal ideation? What is the evidence?. Psychol Med.

[39] Fahy TA (1989). The police as a referral agency for psychiatric emergencies—a review. Med Sci Law.

[40] Webermann AR, Brand BL (2017). Mental illness and violent behavior: the role of dissociation. Borderline Personal Disord Emot Dysregul.

